# CIP2A as a Key Regulator for AKT Phosphorylation Has Partial Impact Determining Clinical Outcome in Breast Cancer

**DOI:** 10.3390/jcm11061610

**Published:** 2022-03-14

**Authors:** Melani Luque, Ion Cristóbal, Marta Sanz-Álvarez, Andrea Santos, Sandra Zazo, Pilar Eroles, Oriol Arpí, Ana Rovira, Joan Albanell, Juan Madoz-Gúrpide, Jesús García-Foncillas, Federico Rojo

**Affiliations:** 1 Pathology Department, IIS-Fundación Jiménez Diaz-UAM, 28040 Madrid, Spain; melani.luque@quironsalud.es (M.L.); marta.sanza@quironsalud.es (M.S.-Á.); szazo@fjd.es (S.Z.); jmadoz@fjd.es (J.M.-G.); 2 Cancer Unit for Research on Novel Therapeutic Targets, Oncohealth Institute, ISS-FJD-UAM, 28040 Madrid, Spain; andrea.santos@quironsalud.es; 3 Translational Oncology Division, Oncohealth Institute, IIS-Fundación Jiménez Diaz-UAM, 28040 Madrid, Spain; jesus.garciafoncillas@oncohealth.eu; 4 Institute of Health Research INCLIVA, 46010 Valencia, Spain; pilar.eroles@uv.es; 5 Medical Oncology Department, Hospital del Mar, 08003 Barcelona, Spain; oarpi@imim.es (O.A.); arovira@imim.es (A.R.); 96087@parcdesalutmar.cat (J.A.)

**Keywords:** p-AKT, CIP2A, prognosis, therapy, early breast cancer

## Abstract

Together with its reported ability to modulate AKT phosphorylation (p-AKT) status in several tumor types, the oncoprotein CIP2A has been described to induce breast cancer progression and drug resistance. However, the clinical and therapeutic relevance of the CIP2A/AKT interplay in breast cancer remains to be fully clarified. Here, we found high p-AKT levels in 80 out of 220 cases (36.4%), which were associated with negative estrogen receptor expression (*p* = 0.049) and CIP2A overexpression (*p* < 0.001). Interestingly, p-AKT determined substantially shorter overall (*p* = 0.002) and progression-free survival (*p* = 0.003), and multivariate analyses showed its CIP2A-independent prognostic value. Moreover, its clinical relevance was further confirmed in the triple negative and HER2-positive subgroups after stratifying our series by molecular subtype. Functionally, we confirmed in vitro the role of CIP2A as a regulator of p-AKT levels in breast cancer cell lines, and the importance of the CIP2A/AKT axis was also validated in vivo. Finally, p-AKT also showed a higher predictive value of response to doxorubicin than CIP2A in ex vivo analyses. In conclusion, our findings suggest that CIP2A overexpression is a key contributing event to AKT phosphorylation and highlights the CIP2A/AKT axis as a promising therapeutic target in breast cancer. However, our observations highlight the existence of alternative mechanisms that regulate AKT signaling in a subgroup of breast tumors without altered CIP2A expression that determines its independent value as a marker of poor outcome in this disease.

## 1. Introduction

Breast cancer is a very heterogeneous disease at the molecular level and it represents the most frequently diagnosed cancer in women worldwide [1,2]. In fact, five different breast cancer subtypes have been defined based on an immunohistochemical criteria that includes expression of estrogen (ER) and progesterone receptors (PR) and human epidermal growth factor receptor 2 (HER2). In the last years, the inclusion of hormonal therapies and other novel treatments (i.e., trastuzumab for HER2 subtype) have notably improved patient prognosis. However, in many cases, outcomes are still very poor due to the appearance of resistances or the absence of more specific and effective treatments for the triple negative (TN) subtype, which includes those cases with worst prognosis [3,4,5].

Aberrant activation of the AKT signaling has been largely associated with human cancer progression and the development of resistance to standard chemotherapies [6,7]. However, the prognostic value of AKT phosphorylation/activation in breast cancer remains to be fully clarified due to the discrepancies observed in the existing literature. Although a work including available data from electronic databases hasindicated that p-AKT could predict adverse outcome in breast cancer [8], this issue needs to be confirmed with further studies. Furthermore, the CIP2A oncoprotein is a potent inhibitor of the tumor suppressor PP2A which impairs PP2A-dependent c-Myc degradation and modulates important effectors such as mTORC1 or E2F1 [9,10,11]. In fact, functional inhibition of PP2A has been reported as a common alteration in breast cancer and CIP2A overexpression as a key event driving PP2A inactivation in this disease [12]. CIP2A have been described to regulate AKT activation in several tumor cell lines including breast cancer [12,13]. Importantly, the therapeutic value of AKT inhibitors such as MK-2206 in breast cancer has been explored with promising results [14,15,16]. Among its biological effects, CIP2A has been reported to regulate proliferation and apoptosis [17], invasion [18], cell cycle progression [19], and it has been associated with human breast cancer aggressiveness [20]. Moreover, CIP2A has been involved in response to drugs such as bortezomib [21], genistein [22], or doxorubicin [23], and it has been proposed as a potential therapeutic target in breast cancer [24]. At the clinical level, CIP2A overexpression hasbeen reported to predict poor outcome in breast cancer [12,25], with a special relevance in the TN subtype [26,27]. Moreover, CIP2A expression has also been described as a predictive marker of recurrence in tamoxifen-treated breast cancer patients [28]. The high relevance of the CIP2A/AKT interplay has been largely reported in the literature. Thus, several compounds such as cucurbitacin B [29] and everolimus [30] in the ER-positive subtype, together with lapatinib [31], arctigenin [32], and the erlotinib derivative TD52 [33] in the TN subtype, have demonstrated to exert their antitumor properties in breast cancer, downregulating both CIP2A and p-AKT. FTY720 is an FDA-approved immunosuppressant for multiple sclerosis which has shown promising antitumor effects in many tumor types. Interestingly, FTY720 has been reported to activate the tumor suppressor PP2A due to its mechanism of action based on CIP2A downregulation and SET blocking, both potent PP2A endogenous inhibitors [34,35,36].

Altogether, the clinical and therapeutic importance of the CIP2A/AKT axis as well as the relevance of CIP2A as a key AKT regulator and plausible molecular target in breast cancer must be fully clarified, which represents our main objective in this study.

## 2. Materials and Methods

### 2.1. Cell Cultures

BT-474 (ATCC HTB-20) and MDA-MB-231 (ATCC HTB-26) were cultured in DMEM/F12 (Sigma Aldrich, Burlington, MA, USA) supplemented with 10% fetal bovine serum (FBS) (Gibco), 2 mmol/L glutamine (GlutaMAX, Gibco), and 1% penicillin G-streptomycin (Gibco). Both cell lines were grown as monolayers at 37 °C under humidified atmosphere with 5% CO_2_. Cells were purchased from American Type Culture Collection (ATCC) and authenticated (LGC Standards). For transfection experiments, breast cancer cells were seeded in 6-well plates and transfected with 10 μL of Lipofectamine 2000 (Life Technologies, Frederick, MD, USA) and 75 nM of CIP2A-specific siRNAs or 1 μg of plasmidic vectors, designed and synthesized by Dharmacon RNA Technologies (Dharmacon, Lafayette, CO, USA).

### 2.2. Patient Samples

Surgical resection specimens from primary breast tumors were obtained from Parc de Salut Mar Biobank (MARBiobanc, Barcelona, Spain), Fundación Jiménez Díaz Biobank (Madrid, Spain), and Valencia Clinic Hospital Biobank (Valencia, Spain). Tumor specimens from formalin-fixed, paraffin-embedded (FFPE) blocks were retrospectively selected from consecutive breast cancer patients diagnosed between 1998 and 2000, which had the following criteria: infiltrating carcinomas, operable, no neoadjuvant therapy, enough available tissue, and clinical follow-up. Tumor–node–metastasis(TNM) staging was classified using the American Joint Committee on Cancer (AJCC) staging system.

Histological grade was defined according to the Scarff–Bloom–Richardson modified by Elston criteria [37]. ER and PR were determined by immunohistochemistry (IHC) (SP1 and PgR636 clones, respectively; Dako, Carpinteria, CA, USA), establishing positivity criteria in >1% of nuclear tumor staining [38]. HER2 amplification was assayed by FISH (Pathvysion; Abbott Laboratories, Abbott Park, IL, USA) [39]. Ki-67 was studied by IHC (MIB1 clone; Dako) [40]. Clinical data were collected from medical clinical records by oncologists. Representative areas of each tumor were carefully selected, and three tissue cores (1 mm diameter) were obtained using a TMA workstation (T1000 Chemicon).

### 2.3. Western Blot Analysis

Total protein lysates were prepared with RIPA buffer containing protease and phosphatase inhibitors. Protein extracts were clarified, denatured, and subjected to SDS-PAGE and Western blotting. The primary antibodies employed were: rabbit polyclonal anti-CIP2A (Sigma); rabbit monoclonal anti-AKT, rabbit monoclonal anti-pAKT (Cell Signaling Technology); and mouse monoclonal anti-β-actin (Sigma). All antibodies were used at 1:1000 except β-actin, which was used at 1:5000. Proteins were detected with the appropriate secondary antibodies conjugated to peroxidase (HRP, GE Healthcare) by chemiluminescence using Immobilon Crescendo Western HRP substrate (Merck Millipore, Burlington, MA, USA).

### 2.4. Ex Vivo Models

Tissue slices larger than 1.5 cm from fresh surgical specimens of patients newly diagnosed with invasive breast cancer were obtained to add ex vivo doxorubicin (2 µg/mL) and assess molecular effects [41]. The samples were processed in sterile conditions immediately after surgical resection. Incubation was performed in 24-well plates at 37 °C in a constant atmosphere of 5% CO_2_ for 24 h. At 24 h, the specimens were fixed in 10% neutral-buffered formalin for 16 h at room temperature and embedded in paraffin under vacuum conditions. These specimens were assayed for molecular markers as described in the IHC section.

### 2.5. In Vivo Animal Model

All animal work was conducted as per the Barcelona Biomedical Research Park (PRBB) Institutional Animal Care and Scientific Committee guidelines. Briefly, 4- to 6-week old female Balb/C nude mice were subcutaneously inoculated in their flank with 10 × 10^6^ MDA-MB-231 cells mixed with Matrigel, as previously described [42]. Tumor growth was measured twice a week. Mice bearing subcutaneous 100–150 mm^3^ tumors were distributed homogenously into four groups of 10 mice each. The first group received saline vehicle intraperitoneally (i.p.) with no active drugs. In the second group, FTY720 (10 mg/kg in saline) was inoculated i.p. every two days [43]. At the end of the experiment, tumors were harvested and formalin-fixed.

### 2.6. Immunohistochemistry

Immunostainings were performed on tissue sections (3 μm) obtained from FFPE tumors, as previously described [44]. Briefly, heat antigen retrieval was performed in the pH9 EDTA-based buffer (Dako) and slides were incubated with same primary antibodies against CIP2A or p-AKT, followed by appropriate anti-Ig horseradish peroxidase-conjugated polymer (Flex+, Dako). Sections were visualized with 3,3′-diaminobenzidine as a chromogen. All stainings were performed in a Dako Autostainer. Sections incubated with normal non-immunized rabbit immunoglobulins were used as negative controls. As positive control, sections of breast tumor with a known expression of targets were used. Antibody sensitivity was calculated in a range of crescent dilutions of primary antibody (CIP2A: 1:20–1:200, p-AKT: 1:1–1:100). Specificity was confirmed in a set of paired fresh frozen, and FFPE samples were processed by Western blot and IHC. Antigen preservation in tissues was confirmed as assaying for expression of phospho-tyrosine using a monoclonal antibody to tyrosine-phosphorylated proteins (clone 4G10, 1:500, Millipore). A semiquantitative histoscore (Hscore) was calculated by estimation of the percentage of tumor cells positively stained with low, medium, or high staining intensity. The final score was determined after applying a weighting factor to each estimate. The formula used was Hscore = (low%) × 1 + (medium%) × 2 + (high%) × 3, and the results ranged from 0 to 300.

### 2.7. Statistical Analysis

Statistical analyses were performed using SPSS 20 for windows (SPSS Inc., Chicago, IL, USA). Overall survival (OS) was defined as the time from diagnosis to the date of death from any cause or last follow-up. Event-free survival (EFS) was defined as the time from diagnosis until relapse at any location, death, or last follow-up. Kaplan–Meier method and survival comparisons were done with the log-rank test if proportional hazard assumption was fulfilled and Breslow otherwise. The Cox proportional hazards model was adjusted, taking into consideration significant parameters in univariate analysis. A *p*-value less than 0.05 was considered statistically significant. Receiver operating curve (ROC) was used to determine the optimal cutoff point based on progression end point for CIP2A and p-AKT expression, as previously described [45]. This work was carried out in accordance with Reporting Recommendations for Tumor Marker Prognostic Studies (REMARK) guidelines [46].

## 3. Results

### 3.1. Prevalence of p-AKT in Human Breast Cancer and Its Association with Clinical and Molecular Parameters

**Figure 1:**
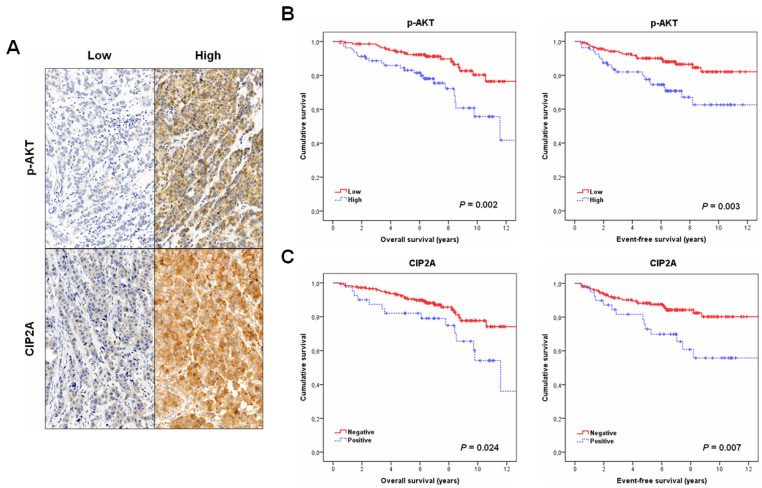
Clinical significance of p-AKT and CIP2A in early breast cancer. (**A**) Immunohistochemical detection of p-AKT and CIP2A showing positive and negative staining. Magnification ×400. Kaplan–Meier analyses of OS and EFS for p-AKT (**B**) and CIP2A (**C**) in a cohort of 220 early breast cancer patients.

We first investigated the prevalence and clinical significance of p-AKT in a cohort of 220 patients with early breast cancer. Patient characteristics are presented in Table S1. Immunohistochemical detections of p-AKT and CIP2A are shown in Figure 1A. p-AKT and CIP2A were expressed mainly in the cytoplasm of cancerous cells.

High p-AKT levels were found in 36.4% of cases (80/220) and were associated with the absence of estrogen receptor expression (*p* = 0.049). Of relevance, p-AKT positively correlated with high CIP2A expression (*p* < 0.001) (Table 1). Furthermore, we also analyzed CIP2A in our cohort, observing high CIP2A levels in 18.2% of cases (40/220), and associated with tumor grade (*p* = 0.042), absence of ER (*p* < 0.001) and PR expression (*p* < 0.001), HER2 amplification (*p* = 0.023), and higher tumor proliferation rates measured using Ki-67 (*p* = 0.033).

Interestingly, high CIP2A expression was found to be associated with HER2 and triple-negative subtypes (*p* = 0.001) and with those patients who relapsed (*p* = 0.001). Associations between CIP2A and clinical and molecular characteristics are shown in Table S2.

### 3.2. Clinical Significance of p-AKT in Human Breast Cancer

Clinical follow-up data were available for all the 220 cases included in the study, with a median of age of 58 years (range: 26–90). Interestingly, the subgroup of patients with high p-AKT showed a substantially shorter OS (*p* = 0.002) and EFS (*p* = 0.003) (Figure 1B). Moreover, we analyzed the prognostic value of CIP2A in our series, observing that CIP2A overexpression was also predictive of worse OS (*p* = 0.024) and EFS (*p* = 0.007) (Figure 1C). We next stratified our series by molecular subtype, observing that p-AKT had prognostic value only in the HER2 (*p* = 0.006 for OS; *p* = 0.004 for EFS) and TN subgroups (*p* = 0.002 for OS; *p* = 0.006 for EFS) (Figure S1). In concordance, CIP2A overexpression determined worse outcome in the HER2 and TN subtypes, but significance was only achieved in the TN subgroup (HER2: *p* = 0.077 for OS; *p* = 0.058 for EFS) (TN: *p* = 0.011 for OS; *p* = 0.010 for EFS) (Figure S2). Finally, multivariate Cox analyses demonstrated that p-AKT is an unfavorable independent factor associated with both OS (Hazard ratio (HR)= 3.25; 95% confidence interval (CI), 1.5–7.2; *p* = 0.003) (Table 2) and EFS (HR = 2.8; 95% CI, 1.3–6.1; *p* = 0.008) (Table S3) in early breast cancer.

### 3.3. Modulation of CIP2A Expression Affects AKT Phosphorylation in Breast Cancer Cells

**Figure 2:**
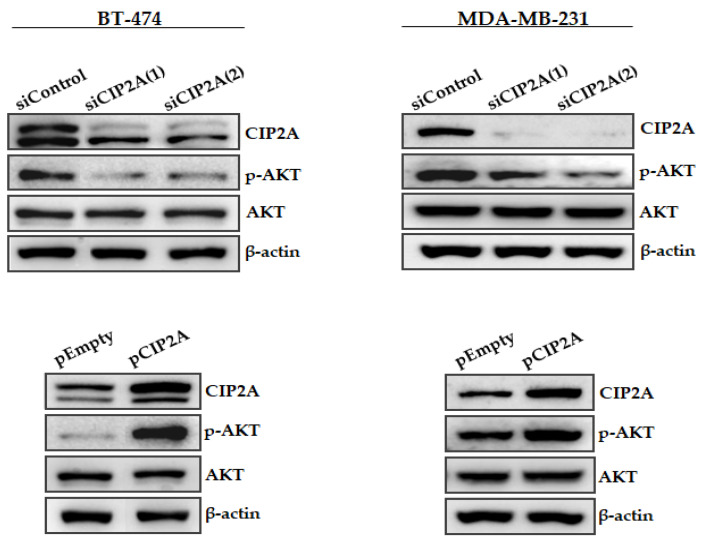
Western blot analysis showing CIP2A and p-AKT expression levels in breast cancer cell lines after ectopic CIP2A modulation.

To confirm the role of CIP2A as a regulator of p-AKT levels in vitro, we ectopically silenced CIP2A using two different specific siRNAs and performed Western blot analyses to evaluate potential changes in p-AKT (Figure 2). Interestingly, we found that CIP2A silencing led to decreased p-AKT levels in both BT-474 and MDA-MB-231 cells. To further confirm these results, we next analyzed the effect of ectopic CIP2A overexpression, observing that, in concordance with the results described above, both cell lines showed increased p-AKT expression. Altogether, these results confirm that CIP2A is involved in the p-AKT regulation ofour breast cancer cell lines.

In order to describe the expression pattern of the CIP2A/p-AKT signaling axis in breast cancer in more detail, and despite the lack of clinical impact in the luminal subgroup, we also investigated the expression levels of both CIP2A and p-AKT in luminal breast cancer cell lines such as MCF-7 and T47D. Of note, we found an expected correlation between CIP2A and p-AKT in both cell lines tested (Figure S3). Furthermore, we analyzed additional downstream markers of the CIP2A/p-AKT signaling. Interestingly, we found changes in c-MYC and p4EBP1 levels in both BT-474 and MDA-MB-231 cells after CIP2A modulation. No differences were observed for pS6 (Figure S4). Finally, we assessed cell proliferation after ectopic modulation of CIP2A in BT-474 and MDA-MB-231 cells. Notably, we found that CIP2A overexpression significantly enhanced cell proliferation in both cell lines. As expected, CIP2A silencing reduced cell growth in both cell lines, but significance was only achieved in the BT-474 cell line (Figure S5).

### 3.4. The Use of FTY720 Inhibits the CIP2A/AKT Axis Leading to Potent In Vivo Antitumor Effects

**Figure 3:**
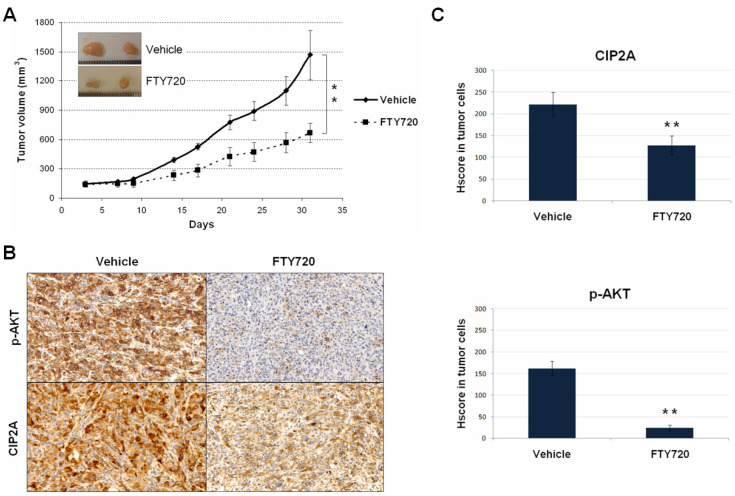
In vivoanalysis of FTY720-mediated CIP2A/AKT inhibition: (**A**) Tumor growth; (**B**) Immunohistochemical detection of p-AKT and CIP2A in tumor samples from control and treated groups. (**C**) Evaluation of differential expression of CIP2A and p-AKT. ** *p* < 0.001.

As statedin the introduction, FTY720 is an FDA-approved immunosuppressant for multiple sclerosis, which has been described to downregulate CIP2A [34,35,36]. Therefore, we performed MDA-MB-231 xenografts that were treated with FTY720 in order to both investigate the potential therapeutic value of the CIP2A/AKT signaling as a novel molecular target in breast cancer and validatein vivo the role of CIP2A as a p-AKT regulator. Interestingly, we observed that the subgroup of mice that received FTY720 treatment showed markedly reduced tumor growth compared with the control subgroup (Figure 3A).

In concordance with the reduced tumor growth detected, we observed that FTY720 significantly reduced proliferation (phosphorylated H3) and enhanced apoptosis (cleaved Caspase 3) (data not shown). Moreover, we carried out immunohistochemical analyses in tumor specimens collected at the end of the experiments to evaluate CIP2A/AKT after FTY720 treatment. As expected, we observed that FTY720 significantly reduced CIP2A expression (Figure 3B). In addition, we also detected a decrease inp-AKT levels, which further suggests the role of CIP2A as an AKT regulator (Figure 3C). Finally, we confirmedin vitrothe antitumor activity of FTY720 in BT-474 and MDA-MB-231 cells, observing that FTY720 led to marked decrease incell growth in both cell lines (Figure S6). Therefore, our results would indicate the potential benefit derived from the use of CIP2A/AKT inhibiting drugs such as FTY720.

### 3.5. AKT Phosphorylation Status Determines Response to Doxorubicin Treatment

**Figure 4:**
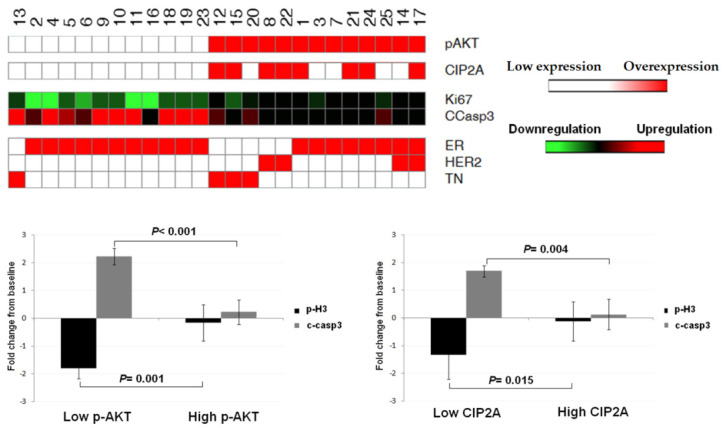
Correlation between p-AKT, CIP2A, phosphorylated H3, and cleaved Caspase 3 in 25 fresh breast cancer specimens treatedex vivowith doxorubicin; pH3: phosphorylated Histone H3; c-casp3: cleaved Caspase 3.

Since CIP2A overexpression has been previously involved in doxorubicin resistance, and since AKT represents a CIP2A downstream effector, we hypothesized that upregulation of p-AKT could play a relevant role determining doxorubicin response in breast cancer patients. Thus, we analyzed p-AKT, CIP2A, phosphorylated H3 (p-H3), and cleaved Caspase 3 (c-casp3) levels in 25 fresh breast cancer specimens with different molecular subtypes treatedex vivowith doxorubicin. Interestingly, our observations showed that p-AKT inversely correlated with proliferation (*p* = 0.001) and apoptosis activation (*p* < 0.001) after doxorubicin treatment. Moreover, 8 out of the 13 cases with high p-AKT levels showed CIP2A overexpression, which was associated with higher proliferation rates (*p* = 0.015) and lower apoptosis (*p* = 0.004) (Figure 4).

Altogether, our findings further support the relevance of CIP2A overexpression as a molecular alteration that leads to p-AKT upregulation, and also indicate that p-AKT plays a predictive role inresponse to doxorubicin in breast cancer patients.

## 4. Discussion

In the last years, it has been progressively reported that CIP2A overexpression plays an oncogenic role and predicts adverse outcomes in a wide variety of human cancers including breast cancer [47,48]. Of relevance, the regulation of AKT phosphorylation status has emerged as one of the key downstream targets modulated by the action of CIP2A [12,13,49,50,51], as we have further confirmed here bothin vitro(Figure 2) andin vivo(Figure 3). We aimed here to study the functional and clinical value of the CIP2A/p-AKT interplay, analyzing the contribution of CIP2A and p-AKT separately. Thus, we investigated both p-AKT and CIP2A levels in a cohort of 220 breast cancer patients, observing that high p-AKT levels strongly correlated with high CIP2A expression and that both markers were predictive of poor outcome. However, multivariate analyses showed that only p-AKT has an independent prognostic value in breast cancer. These results suggest that high CIP2A expression would represent a relevant contributing mechanism to activate AKT signaling, but it is p-AKT upregulation that is the key event determining patient outcomes. The fact that high CIP2A levels were found to be associated with advanced tumor grade and relapse could be indicativethat is a secondary alteration that causes or reinforces an existing p-AKT upregulation, which is in concordance with previous studies highlighting that CIP2A confers aggressivity in breast tumors [20]. Of importance, it has been reported that serum CIP2A levels positively associate with poor prognosis and aggressive breast cancer phenotypes [52], and it could also be of interest to analyze serum p-AKT levels in forthcoming studies.

The existence of a CIP2A-AKT feedback loop in which AKT activation would be mediating a CIP2A upregulation in breast cancer cellshas been reported [53]. However, our clinical findings here show that only 41% of cases (33 out of 80) with high p-AKT had a concomitant CIP2A overexpression (Table 1), and 5 out of 13ex vivomodels with high p-AKT did not show an altered CIP2A expression. These observations suggest that p-AKT upregulation would not be a key regulatory event to determine CIP2A overexpression, but would be a contributing alteration, together with other regulatory drivers.

In fact, the potential relevance of the endogenous inhibitor SET as a regulator of both ERK and AKT signaling [54] has been proposed, indicating that could be interesting to analyze the expression status of SET in those cases with high p-AKT levels and a lack of CIP2A upregulation. To further suggest the potential role of SET regulating the CIP2A/AKT axis, it has been described that the ectopic SET modulation induced changes in both CIP2A and p-AKT levels in TNBC cells [55].

Furthermore, our results indicate that CIP2A is a key determinant of p-AKT in breast cancer, since 82.5% of CIP2A-overexpressing cases also had high p-AKT levels (Table 1). Of note, the absence of CIP2A overexpression in some cases with high p-AKT highlights the existence of alternative molecular mechanisms that contribute to p-AKT in this disease. Breast cancer classification discriminates tumor subtypes with different outcomes and therapeutic implications [3]. When we stratified our cohort by the molecular subtype, we found that p-AKT predicted outcome only in the HER2 and TN subgroups. Despite p-AKT showing similar prevalence in all molecular subtypes, CIP2A was significantly associated with the HER2 and TN subgroups. Altogether, these findings would suggest that CIP2A overexpression confers additional alterations to the activation of AKT signaling that contributes to disease progression. In this line of thinking, we hypothesized that breast cancer patients could benefit from the use of pharmacologic CIP2A/AKT inhibitors such as FTY720. In fact, our promising results showed by in vivo analyses using FTY720 would support that the CIP2A/AKT axis represents a novel therapeutic target in breast cancer.

As CIP2A has been reported to be involved in doxorubicin sensitivity [23], and that p-AKT represents a CIP2A downstream effector, we performedex vivoanalyses to investigate whether p-AKT could be involved in the appearance of resistance to doxorubicin in breast cancer. Interestingly, p-AKT showed a significant predictive value inresponse to doxorubicin treatment, further highlighting its clinical usefulness in this disease. Of note, all eight cases with CIP2A overexpression showed high p-AKT levels (Figure 4). The presence of five cases without CIP2A overexpression and high p-AKT confirmed our previous observations in the cohort of 220 cases about the existence of other molecular mechanisms distinct fromCIP2A involved in p-AKT regulation in breast cancer. Thus, potential pAKT regulators with previously reported relevance in breast cancer such as PDK1 or PTEN should be investigated in forthcoming studies [56]. In addition, PP2A directly targets pAKT, and endogenous PP2A inhibitors such as SET havebeen described to be altered in breast cancer and involved in pAKT regulation [12,54].

Furthermore, these five cases of p-AKT+/CIP2A- showed poor response to doxorubicin, which suggests that it is p-AKT, and not CIP2A, that is crucial in determining doxorubicin sensitivity. Moreover, the association of the CIP2A/AKT axis with doxorubicin response in ex vivo models is in concordance with a previous study showing that CIP2A silencing overcomes doxorubicin resistance in ER-positive breast cancer cells [57]. In addition, it could be relevant to analyze whether those cases with high p-AKT levels could benefit from the use of PP2A activators such as FTY720 in other subtypes than ER-positive, which also inhibits PP2A inhibitors such as CIP2A and SET. Another potential useful strategy could be based on the use of micropeptides following the research line of a recent work using a CIP2A binding peptide with promising results in TNBC cells [58]. In conclusion, our findings support novel therapeutic possibilities for a wide-spectrum of CIP2A/AKT-inhibiting drugs such asFTY720 in breast cancer to be used alone or in combination with standard anthracycline-based chemotherapy.

## Figures and Tables

**Table 1:** Association between p-AKT and clinical and molecular parameters in 220 early breast cancer patients.

	No. Cases	No. Low p-AKT (%)		No. High p-AKT (%)		*p*-Value
p-AKT	220	140	(63.6)	80	(36.4)	
T	220	140		80		0.149
1	107	68	(63.6)	39	(36.4)	
2	89	52	(58.4)	37	(41.6)	
3	22	18	(81.8)	4	(18.2)	
4	2	2	(100)	0	(0)	
N	220	140		80		0.075
0	128	82	(64.1)	46	(35.9)	
1	49	26	(53.1)	23	(46.9)	
2	25	21	(84)	4	(16)	
3	18	11	(61.1)	7	(38.9)	
Stage	218	140		78		0.757
1	80	51	(63.8)	29	(36.2)	
2	96	60	(62.5)	36	(37.5)	
3	42	29	(69)	13	(31)	
Grade	220	140		80		0.060
1	33	15	(45.5)	17	(54.5)	
2	103	68	(66)	30	(34)	
3	84	57	(67.9)	26	(32.1)	
Morphological type	98	52		46		0.063
IDC	93	47	(50.5)	46	(49.5)	
ILC	5	5	(100)	0	(0)	
ER	220	140		80		0.049
Negative	83	46	(55.4)	37	(44.6)	
Positive	137	94	(68.6)	43	(31.4)	
PR	220	140		80		0.398
Negative	99	60	(60.6)	39	(39.4)	
Positive	121	80	(66.1)	41	(33.9)	
HER2	220	140		80		0.252
Negative	149	91	(61.1)	58	(38.9)	
Positive	71	49	(69)	22	(31)	
Hormonal status	213	134		79		0.079
Premenopausal	58	42	(72.4)	16	(27.6)	
Postmenopausal	155	92	(59.4)	63	(40.6)	
Relapse	220	140		80		0.317
No	160	105	(65.6)	55	(34.4)	
Yes	60	35	(58.3)	25	(41.7)	
Ki-67	220	140		80		0.646
Low	147	92	(62.6)	55	(37.4)	
High	73	48	(65.8)	25	(34.2)	
Molecular subtype	220	140		80		0.192
Luminal	95	62	(65.3)	33	(34.7)	
HER2-positive	71	49	(69)	22	(31)	
Triple-negative	54	29	(53.7)	25	(46.3)	
CIP2A	220	140		80		<0.001
Negative	180	133	(73.9)	47	(26.1)	
Positive	40	7	(17.5)	33	(82.5)	

IDC: invasive ductal carcinoma; ILC: invasive lobular carcinoma; ER: estrogen receptor; PR: progesterone receptor.

**Table 2:** Univariate and multivariate Cox analyses in the cohort of 220 patients with early breast cancer.

	Univariate OS Analysis				Multivariate OS Cox Analysis			
	HR	95% CI		*p*-Value	HR	95% CI		*p*-Value
		Lower	Upper			Lower	Upper	
Stage				0.003				0.574
1–2	1.000				1.000			
3	1.951	1.262 to 3.017			0.800	0.367 to 1.742		
Grade				0.029				0.032
1–2	1.000				1.000			
3	1.742	1.060 to 2.864			1.845	1.053 to 3.231		
T				0.001				0.045
1–2	1.000				1.000			
3–4	1.938	1.297 to 2.897			1.884	1.014 to 3.502		
N				<0.001				0.037
−	1.000				1.000			
+	1.656	1.270 to 2.160			1.454	1.023 to 2.067		
CIP2A				0.019				0.746
Low	1.000				1.000			
High	2.154	1.132 to 4.099			0.872	0.381 to 1.995		
p-AKT				0.002				0.003
Low	1.000				1.000			
High	2.587	1.406 to 4.759			3.251	1.477 to 7.158		

## Data Availability

Data sharing is not applicable for this article.

## Supplementary Materials

The following supporting information can be downloaded at: https://www.mdpi.com/article/10.3390/jcm11061610/s1, Figure S1: Kaplan–Meier analyses showing the clinical impact of pAKT in overall (A) and event-free survival (B) in patients with breast cancer and luminal, HER2-positive or triple negative subtypes; Figure S2: Kaplan–Meier analyses showing the clinical impact of CIP2A in overall (A) and event-free survival (B) in patients with breast cancer and luminal, HER2-positive or triple negative subtypes; Figure S3: Western blot analysis showing CIP2A and p-AKT expression levels in the luminal breast cancer cell lines T47D and MCF-7; Figure S4: Western blot analysis showing the status of CIP2A/pAKT downstream effectors after ectopic modulation of CIP2A; Figure S5: Cell growth assay in BT-474 and MDA-MB-231 cells after CIP2A silencing or overexpression. * *p* < 0.05, ** *p* < 0.001; Figure S6: Cell growth assay in BT-474 and MDA-MB-231 cells after FTY720 treatment (10 μM). * *p* < 0.05, ** *p* < 0.001; Table S1: Clinical and molecular characteristics of a series of 220 patients with early breast cancer, Table S2: Association between CIP2A and clinical and molecular parameters in 220 early breast cancer patients, Table S3: Univariate and multivariate Cox analyses in the cohort of 220 patients with early breast cancer.
