# Supplementary material for: CIP2A as a Key Regulator for AKT Phosphorylation Has Partial Impact Determining Clinical Outcome in Breast Cancer
Source: J Clin Med. 2022 Mar 14;11(6):1610. doi: 10.3390/jcm11061610 (PMC8955826; doi:10.3390/jcm11061610)
Supplement: Supplementary file 1 [file jcm-11-01610-s001.zip › Table S1.pdf]

Table S1. Clinical and molecular characteristics of a series of 220 patients with early breast cancer.

		No. (%)	
Age			
	58 (26-90)		
T			
	1	107	(48.6)
	2	89	(40.5)
	3	22	(10)
	4	2	(0.9)
N			
	0	128	(58.2)
	1	49	(22.3)
	2	25	(11.3)
	3	18	(8.2)
Stage			
	1	80	(36.7)
	2	96	(44)
	3	42	(19.3)
Grade			
	1	33	(15)
	2	103	(46.8)
	3	84	(38.2)
Morphological type			
	IDC	93	(94.9)
	ILC	5	(5.1)
ER			
	Negative	83	(37.7)
	Positive	137	(62.3)
PR			
	Negative	99	(45)
	Positive	121	(55)
HER2			
	Negative	149	(67.7)
	Positive	71	(32.3)
Hormonal status			
	Premenopausal	58	(27.2)
	Postmenopausal	155	(72.8)
Chemotherapy			
	None	52	(23.6)
	Adjuvant	145	(65.9)
	Neoadjuvant	23	(10.5)
Hormone therapy			
	No	80	(38.5)
	Yes	128	(134)
Relapse			
	No	160	(72.7)
	Yes	60	(27.3)
Ki-67			
	Low	147	(66.8)

	High	73	(33.2)
Molecular subtype			
	Luminal	95	(43.2)
	HER2-positive	71	(32.3)
	Triple-negative	54	(24.5)
