# Supplementary material for: CIP2A as a Key Regulator for AKT Phosphorylation Has Partial Impact Determining Clinical Outcome in Breast Cancer
Source: J Clin Med. 2022 Mar 14;11(6):1610. doi: 10.3390/jcm11061610 (PMC8955826; doi:10.3390/jcm11061610)
Supplement: Supplementary file 1 [file jcm-11-01610-s001.zip › Table S2.pdf]

Table S2. Association between CIP2A and clinical and molecular parameters in 220 early breast cancer patients.

	No. Cases	No. CIP2A- (%)		No. CIP2A+ (%)		*p*
CIP2A	220	180 (81.8)		40 (18.2)		
T	220	180		40		0.284
1	107	90	(84.1)	17	(15.9)	
2	89	68	(76.4)	21	(23.6)	
3	22	20	(90.9)	2	(9.1)	
4	2	2	(100)	0	(0)	
N	220	180		40		0.304
0	128	108	(84.4)	20	(15.6)	
1	49	37	(75.5)	12	(24.5)	
2	25	22	(88)	3	(12)	
3	18	13	(72.2)	5	(27.8)	
Stage	218	179		39		0.052
1	80	70	(87.5)	10	(12.5)	
2	96	72	(75)	24	(25)	
3	42	37	(88.1)	5	(11.9)	
Grade	220	180		40		**0.042**
1	33	30	(90.9)	3	(9.1)	
2	103	88	(85.4)	15	(14.6)	
3	84	62	(73.8)	22	(26.2)	
Morphological type	98	84		14		0.591
IDC	93	79	(84.9)	14	(15.1)	
ILC	5	5	(100)	0	(0)	
ER	220	180		40		**<0.001**
Negative	83	57	(68.7)	26	(31.3)	
Positive	137	123	(89.8)	14	(10.2)	
PR	220	180		40		**<0.001**
Negative	99	71	(71.7)	28	(28.3)	
Positive	121	109	(90.1)	12	(9.9)	
HER2	220	180		40		**0.023**
Negative	149	128	(85.9)	21	(14.1)	
Positive	71	52	(73.2)	19	(26.8)	
Hormonal status	213	173		40		0.125
Premenopausal	58	51	(87.9)	7	(12.1)	
Postmenopausal	155	122	(78.7)	33	(21.3)	
Relapse	220	180		40		**0.001**
No	160	139	(86.9)	21	(13.1)	
Yes	60	41	(68.3)	19	(31.7)	
Ki-67	220	180		40		**0.033**
Low	147	126	(85.7)	21	(14.3)	
High	73	54	(74)	19	(26)	
Molecular subtype	220	180		40		**<0.001**
Luminal	95	90	(94.7)	5	(5.3)	
HER2-positive	71	52	(73.2)	19	(26.8)	
Triple-negative	54	38	(70.4)	16	(29.6)	

p-AKT	220	180		40		**<0.001**
Low	140	133	(95)	7	(5)	
High	80	47	(58.8)	33	(41.2)	

IDC: invasive ductal carcinoma; ILC: invasive lobular carcinoma; ER: estrogen receptor; PR: progesterone receptor.
