# Supplementary material for: CIP2A as a Key Regulator for AKT Phosphorylation Has Partial Impact Determining Clinical Outcome in Breast Cancer
Source: J Clin Med. 2022 Mar 14;11(6):1610. doi: 10.3390/jcm11061610 (PMC8955826; doi:10.3390/jcm11061610)
Supplement: Supplementary file 1 [file jcm-11-01610-s001.zip › Table S3.pdf]

Table S3. Univariate and multivariate Cox analyses in the cohort of 220 patients with early breast cancer.

	Univariate EFS analysis				Multivariate EFS Cox analysis			
		95% CI				95% CI		
	HR	Lower	Upper	Significance	HR	Lower	Upper	Significance
Stage				**0.001**				0.450
1-2	1.000				1.000			
3	2.010	1.309 to 3.086			0.735	0.331 to 1.632		
Grade				**0.018**				**0.043**
1-2	1.000				1.000			
3	1.805	1.104 to 2.951			1.773	1.017 to 3.090		
T				**0.001**				**0.033**
1-2	1.000				1.000			
3-4	1.964	1.329 to 2.904			1.968	1.056 to 3.668		
N				**<0.001**				**0.014**
-	1.000				1.000			
+	1.707	1.311 to 2.223			1.570	1.098 to 2.245		
CIP2A				**0.009**				0.743
Low	1.000				1.000			
High	2.345	1.234 to 4.456			1.142	0.516 to 2.529		
p-AKT				**0.004**				**0.008**
Low	1.000				1.000			
High	2.440	1.325 to 4.494			2.813	1.306 to 6.059		
